# Clinical Outcomes of Classical, V-Y, and Z-plasty Frenectomies: A Triad of Techniques

**DOI:** 10.7759/cureus.94262

**Published:** 2025-10-10

**Authors:** Roopse Singh, Nancy Saxena, Aishwarya Tripathy, Nitin Tomar, Mayur Kaushik

**Affiliations:** 1 Periodontology, Subharti Dental College and Hospital, Meerut, IND; 2 Periodontology, Lokpriya Hospital, Meerut, IND; 3 Periodontology and Implantology, Subharti Dental College and Hospital, Meerut, IND

**Keywords:** dental surgery, frenectomy, frenum, periodontal health, perio plastic surgery

## Abstract

The frenum is a fold of mucous membrane that connects the gingiva, underlying periosteum, and alveolar mucosa to the lip and cheek. It can negatively impact periodontal health by impeding plaque control or causing muscle pull if it is placed too near the gingival margin. An aberrant frenum in the maxillary region can also cause aesthetic issues or impede the results of orthodontic treatment, especially when midline diastema is present. This frequently leads to relapse following treatment. Frenectomy is the method used to manage such abnormal frenal attachments. This case series demonstrates various frenectomy techniques, including conventional (classical) frenectomy, V-Y plasty, and Z-plasty.

## Introduction

The desire for an ideal smile has driven an increasing number of people to seek dental care to meet aesthetic demands. Maxillary labial frenum is considered one of the common etiological factors for persistent midline diastema, and its presence poses a significant aesthetic concern in adults that requires careful clinical attention. In a study [1], Priyanka et al. found that frenal attachments play a crucial role in maintaining oral health and esthetics, and abnormal variations often necessitate surgical correction.

In a study [2], Dibart et al. found that the frenum is a fold of mucous membrane that contains muscle fibers of the gingiva, underlying periosteum, and alveolar mucosa to the lips and cheeks. The maxillary labial frenum usually has a triangular shape. As a dynamic structure, it undergoes significant changes in size, shape, and location as it grows and develops. The frenum is typically thick and broad in early childhood, but as growth progresses, it gets thinner and smaller. Management strategies such as frenotomy or frenectomy are determined based on the level of attachment and functional interference.

According to a study done by [3], Shivanni, et al. found that the prevalence of 22.3% midline diastema in adult orthodontic patients; high frenal attachment was the most common etiologic factor. However, in some instances, this infantile pattern-which is frequently linked to hypertrophic frenum and high coronal attachment-remains. This normal structure can occasionally appear as a wide, fibrous band that affects oral hygiene, lip mobility, recession, and general appearance, all of which can lead to the development of diastema. Additionally, frenal with coronal attachment may make gingival recession more likely by preventing efficient toothbrushing or by pulling on the muscles that open the gingival sulcus. The labial frenum is a significant factor in diastema persistence when it exerts tension on the interdental papilla.

In a study [4], Devishree et al. found that the ectolabial bands, which join the palatine papilla and the tubercle of the upper lip, are the post-eruptive remnant of the maxillary labial frenum. An abnormal frenal attachment and a V-shaped bony cleft develop when the maxillary central incisors erupt too widely apart, preventing bone formation beneath the frenum. When the frenum of the mandible is linked to decreased vestibular depth and inadequate gingival width, it is considered abnormal.

In a study [5], Khan et al. found that clinical diagnosis of abnormal frenum can be made by applying tension to the frenum and monitoring the blanching effect brought on by localized ischemia or the movement of the interdental papilla. When a frenum appears too wide, when there is no visible band of attached gingiva along the midline, or when the frenum's extension pushes the interdental papilla aside, it is deemed pathogenic. Diode laser frenectomy has been reported to achieve predictable esthetic outcomes with less bleeding and faster healing. In various studies [6-7], Archer et al. found that classical methods described in surgical atlases have long served as the foundation of frenectomy procedures.

The frenal attachment categorizes frenal attachments based on the location of fiber insertion as mucosal type, in which fibers attach at the mucogingival junction, while in the gingival type, the fibers insert into the attached gingiva. The papillary type is characterized by fibers extending into the interdental papilla. In a study [4], Khan et al. found that the papilla-penetrating type, in which the fibers pass through the alveolar process and reach the palatine papilla.

The two treatment options for managing aberrant frena are frenectomy and frenotomy, which can be performed using two modalities: conventional or thermal, the latter including electrosurgery and laser. In a study [3], Shivanni et al. found that frenectomy entails the frenum's total excision, including its attachment to the bone beneath. It is frequently recommended when there is an irregular midline diastema. In a study by Dibart et al. [2], the term *frenotomy* describes the incision and movement of the frenal attachment. As it realigns the frenum to produce a sufficient zone of attached gingiva, it is usually adequate for periodontal purposes.

## Case presentation

Three patients reported to the Department of Periodontology, Subharti Dental College and Hospital, with the chief complaint of spacing in the upper anterior region. On clinical examination, all three patients presented with aberrant frenal attachment, and the tension test was found to be positive. Based on the clinical findings, frenectomy procedures were planned. The surgical techniques chosen varied according to the type and extent of the frenal attachment. The entire procedure was explained to each patient, and written informed consent was obtained before surgery.

Case 1: Conventional (classical) frenectomy

**Figure 1:**
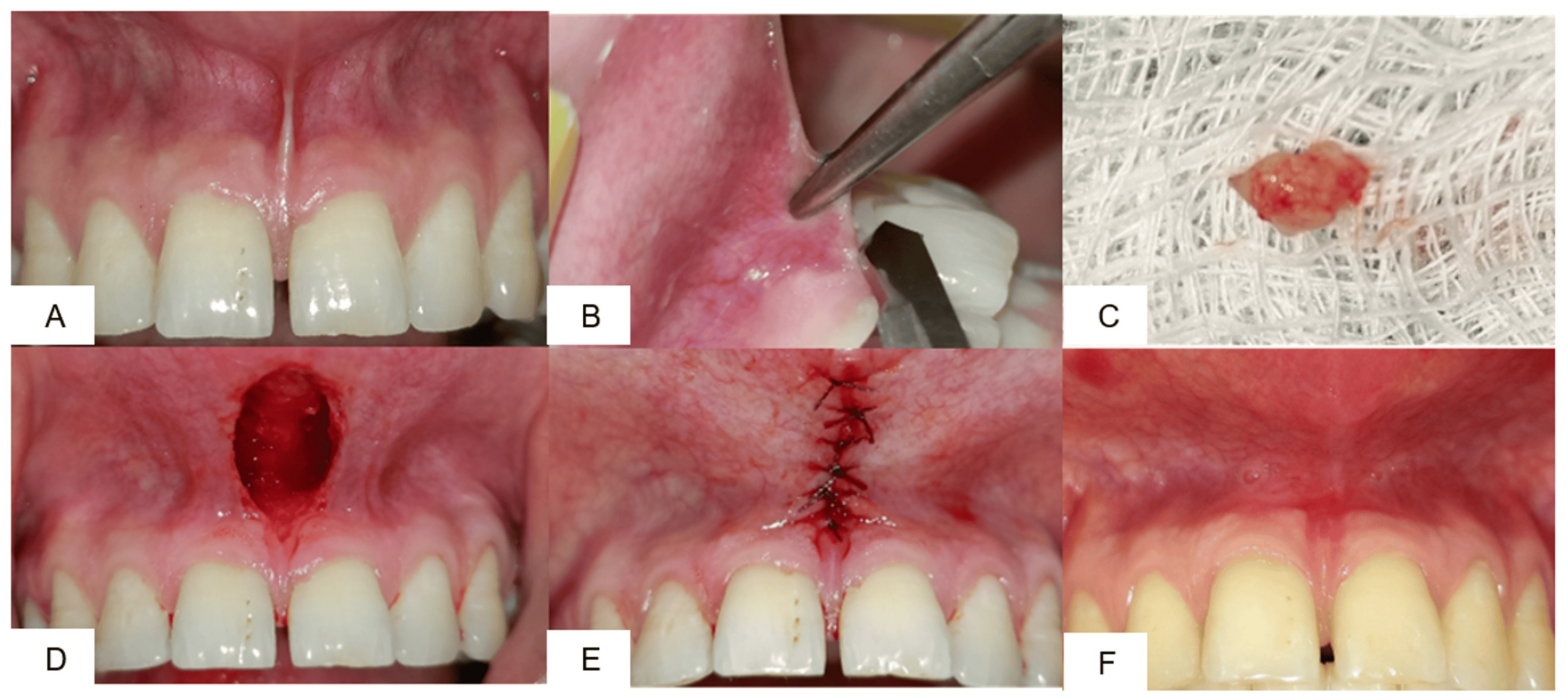
(A) preoperative view; (B) frenum held with hemostat; (C) excised frenum; (D) separation of attached fibers; (E) placement of 5-0 sutures; (F) one-month postoperative view.

A 24-year-old male patient with a broad frenum was selected for a conventional frenectomy (Figure 1A). After administration of local anesthesia (2% lignocaine with 1:80,000 adrenaline), a hemostat was inserted into the depth of the vestibule engaging the frenum (Figure 1B). Using a No. 15 Bard-Parker blade, two incisions were made - one above and one below the hemostat. The triangular segment of the frenum was excised (Figure 1C), and the underlying fibrous attachments were carefully dissected. The wound area was left in a rhomboidal shape (Figure 1D) and partially sutured with 5-0 resorbable suture (Figure 1E). The patient was recalled after 10 days for suture removal. Healing was satisfactory, and a 1-month follow-up showed a reduction in frenal tension with no functional disturbance (Figure 1F).

Case 2: V-Y plasty

**Figure 2:**
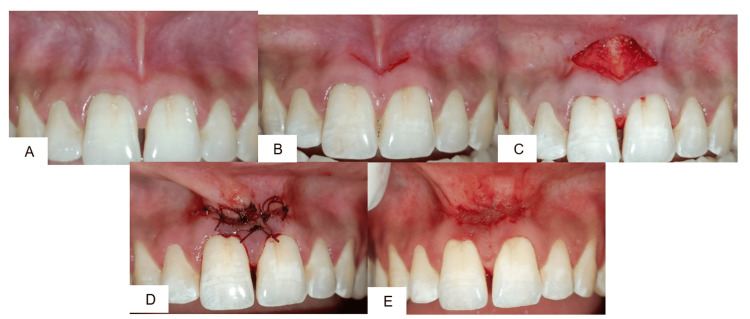
(A) Preoperative view; (B) V-shaped incision; (C) separation of attached fibers; (D) placement of 5-0 sutures in Y shape; (E) one-month postoperative view.

A 27-year-old male patient with a broad frenum extending into the interdental region underwent frenectomy by V-Y plasty (Figure 2A). Following local anesthesia, a V-shaped incision was placed (Figure 2B), and the frenum was repositioned apically (Figure 2C). During suturing, the incision line was converted into a Y configuration to relieve tension and allow for apical repositioning. Sutures were placed (Figure 2D). Sutures were removed after one week. On recall at 10 days, healing was uneventful, and the vestibular depth was well maintained. At one-month follow-up, the diastema showed stability, and the patient reported no discomfort in speech or mastication (Figure 2E).

Case 3: Z-plasty

**Figure 3:**
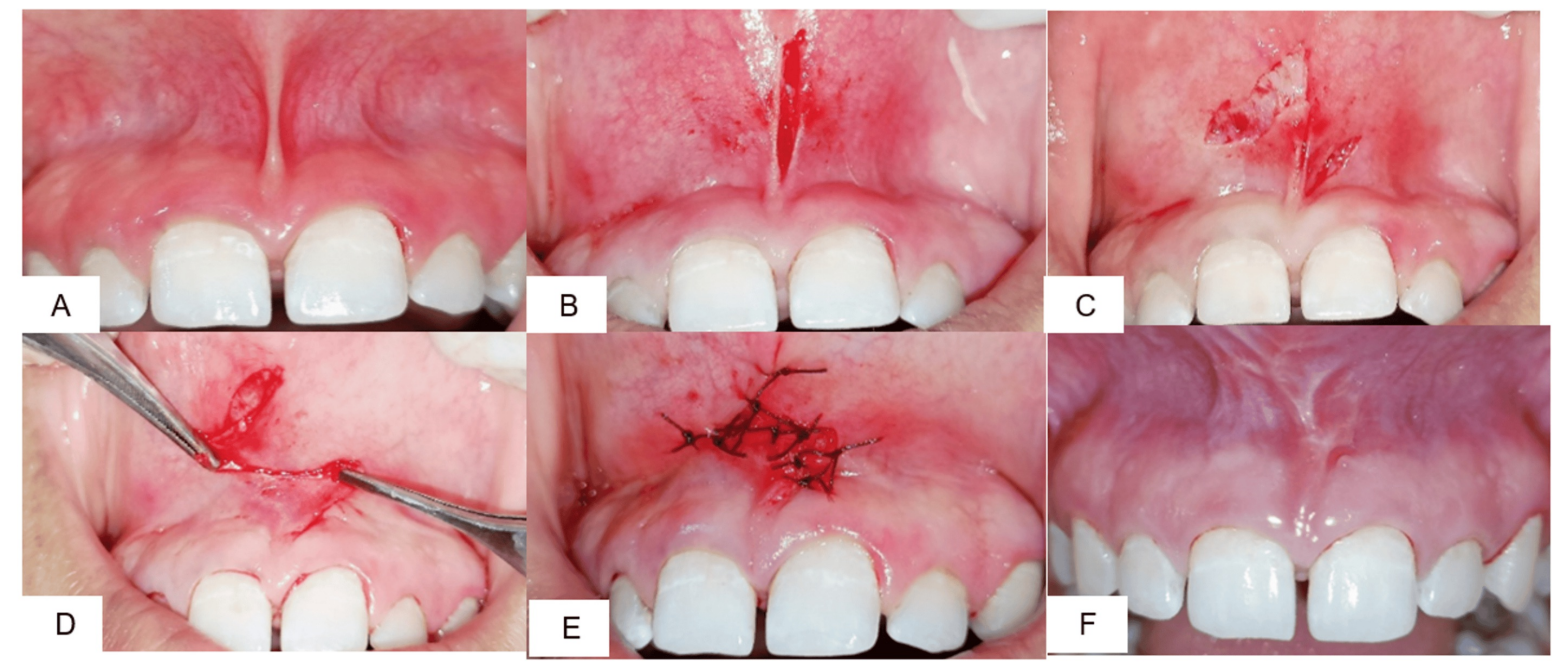
(A) Preoperative view; (B) elliptical incision; (C) pedicle 1 and pedicle 2; (D) Z-plasty; (E) placement of 5-0 sutures in Z shape; (F) one-month postoperative view.

A 25-year-old male patient with hypertrophic frenum (Figure 3A) and shallow vestibule was treated with Z-plasty. After infiltration with local anesthesia, the frenum was incised (Figure 3B) and two oblique limbs at approximately 60°-70° were created (Figure 3C). Double rotation flaps were raised and transposed to achieve a 90° shift, in Z shape, effectively closing the incision horizontally (Figure 3D). The flaps were sutured with 5-0 resorbable suture (Figure 3E). Sutures were removed after seven days. Healing was satisfactory, with a good aesthetic outcome, and vestibular depth was increased. At one month, no recurrence of frenal pull was seen (Figure 3F).

In all three patients, postoperative healing was uneventful, and the overall gingival and soft tissue appearance was healthy and esthetic. Patients were referred to the Department of Orthodontics for further management of midline diastema.

Three patients with aberrant frena underwent different surgical techniques. In Case 1, a 24-year-old male was treated with conventional frenectomy, where the frenum was excised using a hemostat and blade, followed by partial suturing; healing at one month was satisfactory with reduced tension. In Case 2, a 27-year-old male underwent V-Y plasty; the frenum was repositioned apically with a V incision converted to a Y during suturing, resulting in stable diastema and good vestibular depth at one month. In Case 3, a 25-year-old male was managed with Z-plasty, involving oblique incisions and transposition of flaps; this provided increased vestibular depth, esthetic improvement, and no recurrence at one month.

## Discussion

According to a study [8], Sharma et al. found that surgical planning for an aberrant frenum is primarily based on clinical examination, since the diagnosis relies on the position of the frenum, the level of gingival attachment, and the presence of functional or esthetic problems such as midline diastema, gingival recession, or compromised plaque control. Clinical tests such as the blanch test (pull test) are widely accepted for diagnosing abnormal frenal attachments.

Also according to a study [9], Ahn et al. found that radiographs are not routinely necessary for isolated soft-tissue frenum but are indicated when the clinical picture raises suspicious of underlying bony causes for the diastema (supernumerary teeth alveolar cleft, or other osseous defects) or when orthodontic timing/approach would change, in those cases periapical/occlusal films or CBCT (selectively) should be used to exclude bony pathology before definitive surgery. But in our case series, the midline diastema was not that evident, so we planned the surgery on the basis of clinical examination.

According to a study by Devishree et al. [4], the advantages and disadvantages of different surgical approaches are discussed below.

Classical technique

Advantages: Straightforward, widely practiced, cost-effective, and ensures predictable elimination of the frenum when performed adequately.

Disadvantages: Associated with increased intraoperative bleeding, need for sutures, and potentially greater postoperative discomfort. Healing may take longer compared with laser or electrosurgical methods. In addition, central scar formation may occur if vestibular deepening is not incorporated.

Z-plasty

Advantages: Particularly suitable for thick or low frena associated with midline diastema. Allows removal of the fibrous band while simultaneously lengthening the vestibule. The technique reorients the scar line, reduces contracture, and minimizes midline scar tethering, offering favorable aesthetic outcomes.

Disadvantages: Requires advanced surgical skill and precise flap design, involves a longer operative time, and may not be ideal for young or uncooperative patients.

V-Y plasty 

Advantages: Provides additional vestibular deepening and redistributes scar tissue, making it valuable when gingival augmentation or esthetic improvement is also required.

Disadvantages: More invasive than simple excision, necessitates suturing and a higher degree of surgical expertise, and may involve slightly greater short-term morbidity.

In a study [10], Tyagi et al. found that both treatments can be carried out on their own or in conjunction with periodontal therapy. While lingual mandibular involvement is less common, frenal abnormalities are most commonly found in the midline region of maxillary and mandibular central incisors, as well as in canine and premolar areas. Even the conventional *classical* frenectomy technique continues to be performed widely, especially in resource-limited clinical settings.

In a study [11], Chaubey et al. found that classical technique, V-Y plasty, and Z-plasty are some of the variations of the traditional frenectomy that have been developed over time in order to overcome the functional and aesthetic drawbacks of the original method. Perio-esthetic approaches such as combining frenectomy with a laterally positioned flap have been advocated to prevent scar formation and enhance esthetic results.

On the other hand, Z-plasty and V-Y plasty have been considered the method of choice for thick, fibrotic frena with low insertion, especially when associated with midline diastema and shallow vestibule, as it allows both removal of fibrous tissue and vestibular deepening. In a study [4], Devishree et al. also found that the Z-plasty technique was ideal for a broad, thick hypertrophic frenum with a low insertion, which was associated with an inter-incisor diastema and a short vestibule. It achieved both the removal of the fibrous band and the vertical lengthening of the vestibule.

According to a study [12], Brookes et al. reported that readhesion or revision rates after frenotomy/frenectomy vary widely in the literature. It can occur in around 13% of frenectomy/frenotomy procedures, depending on the definition, follow-up length, and study setting. 

When muscle attachments and fibers are not completely removed, the frenum regenerates after a frenectomy. According to a study by Tanik et al. [13], 12% of frena recurred after frenectomy. 

As reported by Awooda [14], the most common reasons for recurrence are incomplete release of fibrous or muscular fibers, inadequate apical repositioning, or residual tension. Therefore, recurrence can be reduced by ensuring complete dissection of the periosteum.

Our study included a one-month follow-up. However, according to Awooda [14], a one-month follow-up is insufficient to judge durability after frenectomy because various case series and case reports show heterogeneous but generally favorable long-term outcomes when complete excision and appropriate apical repositioning are achieved. Also, initial healing may appear satisfactory, but fibrous proliferation or scar tissue remodeling can lead to recurrence over time. So, for long-term stability of the present case series, we are planning a long-term randomized clinical trial with longer follow-ups.

The limitation of this case series is the small sample size and short follow-up period, as only three cases were included, and outcomes were assessed at one month. Therefore, we are planning a long-term randomized clinical trial with a longer follow-up period.

## Conclusions

Frenectomy should be planned not only for functional correction but also for achieving optimal aesthetics. While several surgical options are available, the choice of technique must be tailored to the type of frenal attachment to ensure both functional stability and pleasing esthetic outcomes. In the present case series, papillary frenum was managed using the conventional technique, V-Y plasty, and Z-plasty, which proved simple to perform and yielded satisfactory esthetic and functional results with high patient satisfaction.
